# Effects of *Castanopsis echinocarpa* on Sensorineural Hearing Loss via Neuronal Gene Regulation

**DOI:** 10.3390/nu16162716

**Published:** 2024-08-15

**Authors:** Isabel Rodriguez, Youn Hee Nam, Sung Woo Shin, Gyeong Jin Seo, Na Woo Kim, Wanlapa Nuankaew, Do Hoon Kim, Yu Hwa Park, Hwa Yeon Lee, Xi Hui Peng, Bin Na Hong, Tong Ho Kang

**Affiliations:** 1 Department of Oriental Medicine Biotechnology, Graduate School of Biotechnology, Kyung Hee University, Global Campus, Yongin 17104, Republic of Korea; isabelula3r@gmail.com (I.R.); 01073205620@khu.ac.kr (S.W.S.); seogj115@gmail.com (G.J.S.); wanlapa.nuankaew@gmail.com (W.N.); kdh2@dkpharm.co.kr (D.H.K.); pyh@dkpharm.co.kr (Y.H.P.); lllhy8024@naver.com (H.Y.L.); 2 Invivotec Co., Ltd., Seongnam 13449, Republic of Korea; 01030084217@hanmail.net (Y.H.N.); nawookim@invivotec.com (N.W.K.); 3 Department of Garden, Xishuangbanna Tropical Botanical Garden, Chinese Academy of Sciences, Menglun 666303, China; xihuipeng@xtbg.ac.cn

**Keywords:** sensorineural hearing loss, *Castanopsis echinocarpa*, ototoxicity, noise-induced hearing loss, neuronal function and survival

## Abstract

Sensorineural hearing loss (SNHL), characterized by damage to the inner ear or auditory nerve, is a prevalent auditory disorder. This study explores the potential of *Castanopsis echinocarpa* (CAE) as a therapeutic agent for SNHL. In vivo experiments were conducted using zebrafish and mouse models. Zebrafish with neomycin-induced ototoxicity were treated with CAE, resulting in otic hair cell protection with an EC_50_ of 0.49 µg/mL and a therapeutic index of 1020. CAE treatment improved auditory function and protected cochlear sensory cells in a mouse model after noise-induced hearing loss (NIHL). RNA sequencing of NIHL mouse cochleae revealed that CAE up-regulates genes involved in neurotransmitter synthesis, secretion, transport, and neuronal survival. Real-time qPCR validation showed that NIHL decreased the mRNA expression of genes related to neuronal function, such as *Gabra1*, *Gad1*, *Slc32a1*, *CaMK2b*, *CaMKIV*, and *Slc17a7*, while the CAE treatment significantly elevated these levels. In conclusion, our findings provide strong evidence that CAE protects against hearing loss by promoting sensory cell protection and enhancing the expression of genes critical for neuronal function and survival.

## 1. Introduction

Sensorineural hearing loss (SNHL) is a major global health concern, with its prevalence significantly increasing over the past decade. This rise appears to be correlated with increased exposure to hazardous noise levels in daily life, ototoxic agents, and aging [1]. Currently, more than 466 million people worldwide suffer from hearing loss, which is projected to keep rising. Notably, excessive occupational noise exposure is a critical health hazard, accounting for 16% of adult hearing loss cases, as reported by the World Health Organization [2].

Auditory functions require complex interactions among the sensory cells in the inner ear, specifically involving otic hair cells and spiral ganglion neurons. In noise-induced hearing loss (NIHL), high-frequency sounds damage the cochlear sensory cells, which lack the capacity to regenerate [3]. Therefore, hearing loss prevention strategies are crucial to attenuate cochlear sensory cell loss by blocking several cellular mechanisms that contribute to premature hearing impairment [4,5,6]. Recently, neuroprotective drugs, antioxidants, anti-inflammatory, and anti-apoptotic agents have been widely used to improve hearing deficits in NIHL [7]. Specifically, polyphenols have been shown to protect sensory cells and improve hearing function [8,9]. However, natural products have not been studied extensively as potential treatments. Thus, we have been studying natural products with high polyphenol content as candidates for hearing loss prevention [10,11].

Recently, growing research has focused on herbal species as multi-target approaches to treat sensorineural hearing loss in vitro and in vivo. Currently, clinical trials have been carried out using different traditional herbs, including *Ginkgo biloba*, *Panax ginseng*, and *Astragalus propinquus* [12]. The molecular mechanisms of natural products on protection and recovery from auditory insults involve targeting several pathophysiological states such as oxidative damage, inflammation, disruption of ion homeostasis, pro-apoptotic and apoptotic mediators, and decreased survival regulation. Such a multi-target approach on the inner ear makes natural products important for drug discovery on hearing impairments [12]. We previously demonstrated SNHL amelioration by using natural products. To name a few, *Curculigo orchioides* showed protective efficacy from ototoxicity through a reduction in oxidative stress and increased scavenging activity against free radicals in vitro [13] and improved auditory function on NIHL in vivo [14]; *Scutellaria baicalensis* showed improved auditory function on different models of SNHL in vivo through lipoxygenase inhibition [11]; and avocado oil (*Persea ameriacana*) demonstrated efficacy against SNHL in vitro and in vivo by reducing the altered gene expression related to oxidative stress, cytokine production, and protein synthesis [15].

After extensive screening, we identified *Castanopsis echinocarpa* (CAE)’s potential as a therapy for SNHL. Despite recent interest in Castanopsis species, they have not yet been widely studied. The leaves of these species are reported to be rich in polyphenols, such as galloyl quinic acids, triterpene hexahydroxydiphenoyl esters, ellagitannins, phenol glucosides, condensed tannins, and flavonol glycosides. Some species have already been reported to possess powerful antioxidant properties, effective against oxidative stress and inflammation and preventive against apoptosis [16,17,18,19]. Currently, no studies have investigated Castanopsis species in the context of SNHL, and specifically, CAE has not reported any pharmacological activity. Therefore, our aim is to demonstrate CAE’s efficacy on different models of SNHL in vivo and to elucidate its mode of action.

In this study, we investigated the protective effect of CAE on different models of SNHL in vivo, using the neomycin (NM)-induced ototoxicity model in zebrafish and the NIHL model in mice. CAE demonstrated significant improvement in auditory function and protective effects on auditory structures. To further assess CAE’s mechanism of action, we identified differentially expressed genes in the cochlea of NIHL mice treated with CAE using RNA sequencing. Then, for data validation, we performed real-time quantitative PCR. We found that the protective effect of CAE on SNHL might be explained by the up-regulation of gene expression related to neural functions, such as genes involved in neurotransmitter synthesis, transport and release, and neuronal survival.

Recent research has demonstrated that the loss of hearing function is also related to the damage in synaptic connections among cochlear hair cells and spiral ganglion neurons; such damage can occur much earlier than otic hair cell death [20]. This study thus proposes a therapeutic approach based on recent perspectives to treat hearing impairments.

## 2. Materials and Methods

### 2.1. Plant Collection and Voucher Specimen Information

The plant extract of *Castanopsis echinocarpa* Miq. (FBM070-066) used in this research was obtained from the International Biological Material Research Center at the Korea Research Institute of Bioscience and Biotechnology (Daejeon, Republic of Korea). The plant was collected in Menglun Mengla district, Xishuangbanna, Yunnan province of China, in December 2009. A voucher specimen (KRIB 0062375) is kept in the herbarium of the Korea Research Institute of Bioscience and Biotechnology.

### 2.2. Extraction Procedure for Castanopsis Echinocarpa (CAE)

The raw material was stored at room temperature until extraction. The extraction process comprised two phases. Initially, we placed 1 kg of the aerial parts in a commercial extractor (KS220, Kyungseo E&P Co., Ltd., Incheon, Republic of Korea) with 8 L of 70% ethanol for 2 h. Subsequently, an additional 4 L of 70% ethanol was added, and the extraction continued for another hour. The extract was filtered and concentrated, then frozen at −50 °C for 24 h and freeze-dried in a rotary evaporator (HS-2001NS, Hahanshin Scientific, Gimpo-si, Republic of Korea) for 7 days. The final yield of CAE was 15.5%, and the product was stored at −20 °C.

### 2.3. Animals

Adult zebrafish (*Danio rerio*), wild-type strain, were housed in an S-type 1500(W) × 400(D) × 2050(H) mm system (WoojungBio, Inc., Suwon, Republic of Korea). Spawning and egg collection followed established protocols [15]. Embryos were maintained under a 14 h light/10 h dark cycle at 28.5 °C ± 0.5 until 6 days post-fertilization, after which they were randomly selected for experiments.

Six-week-old male mice, ICR strain, were obtained from Orient Bio, Inc. (Seongnam, Republic of Korea). They were housed under a 12 h light/dark cycle with food and water ad libitum and environmental conditions as follows: 23.0 ± 2.0 °C temperature and 50.0 ± 5.0% humidity. Following a week of acclimation, mice were evaluated by auditory brainstem response (ABR) test to confirm normal hearing (≤25 dB), representing the inclusion criteria as described in previous studies [15].

### 2.4. Ethical Statement

All the procedures involving zebrafish and mice were conducted following protocols approved by the Animal Care and Use Committee of Kyung Hee University [KHUASP-21-230 and KHUASP-21-229, respectively].

### 2.5. Neomycin-Induced Ototoxicity in a Zebrafish Model

Zebrafish larvae were exposed to Neomycin sulfate (MB Cell Co., Irvine, CA, USA) to induce ototoxicity, following established methods [15]. Post-exposure, larvae were first rinsed and then treated with 0.03% sea salt solution and CAE for 6 h at 28 °C, respectively. Post-treatment, larvae were stained with 0.1% YO-PRO-1 (Thermo Fisher Scientific Inc., Waltham, Massachusetts, USA) for 30 min for otic hair cell visualization, anesthetized with 0.04% tricaine, and examined using a fluorescence microscope (Olympus 1 × 70; Olympus Co., Tokyo, Japan). Images were analyzed with Focus Lite software (Focus Co., Daejeon, Republic of Korea).

### 2.6. The 50% Effective Concentration (EC_50_)

Varying concentrations (0.01 to 10 µg/mL) of CAE were defined to treat zebrafish larvae. EC_50_ values were determined by non-linear regression using GraphPad Prism version 5.01 software (Graph Pad Software, San Diego, CA, USA).

### 2.7. The 50% Lethal Concentration (LC_50_)

Varying concentrations (50 to 1000 µg/mL) of CAE were defined to treat zebrafish larvae. LC_50_ values were determined by non-linear regression using GraphPad Prism version 5.01 software (Graph Pad Software, San Diego, CA, USA).

### 2.8. Therapeutic Index (TI)

The therapeutic index (TI) was calculated to define the safety margin of CAE.

TI = LC_50_/EC_50_

### 2.9. Noise-Induced Hearing Loss (NIHL) in Mice

NIHL procedures were performed as previously described [15]. Briefly, mice were exposed to 115 dB sound pressure level (SPL) broadband noise (100 Hz to 10 kHz) for 90 min. Post-exposure, mice were divided into four groups (*n* = 10/group) and treated orally once a day, as follows: control (0.3 mL distilled water), 100 mg/kg (CAE 100), 300 mg/kg (CAE 300), and 500 mg/kg (CAE 500) of CAE in distilled water, starting one day after noise exposure.

### 2.10. Auditory Brainstem Response (ABR) Test

Auditory function was measured using channel recording (Intelligent Hearing Systems, Miami, FL, USA) as previously described [15]. Hearing thresholds were evaluated with ABR using clicks, 8 kHz, and 16 kHz stimuli at 1, 10, and 20 days post-noise exposure.

### 2.11. Evaluation of Otoprotective Effects of Cochlear Hair Cells

After 20 days of drug administration, the cochleae were harvested, fixed, decalcified, and microdissected as described previously [15]. Cochleae were stained for hair cell visualization with 5 U/mL rhodamine phalloidin (Thermo Fisher Scientific Inc., Gainesville, FL, USA), and outer hair cells (OHCs) at the apex, middle, and base of the cochlea were identified and counted in a 1 mm strip. Hair cells were evaluated in three groups: control, NIHL, and NIHL + CAE (*n* = 6 per group) using fluorescence microscopy.

### 2.12. mRNA Sequencing and Pathway Analysis

Twenty days post-administration, the cochleae were harvested for RNA extraction using TRIzol™ reagent (Thermo Fisher Scientific Korea Ltd., Seoul, Republic of Korea) and purified with a RNeasy mini kit (QIAGEN, Hilden, Germany). For sampling, both right and left cochleae were collected from 3 animals in each group. RNA quality and quantity were assessed using an Agilent 2100 Bioanalyzer (Agilent, Santa Clara, CA, USA). Sequencing libraries were prepared with the TruSeq Stranded mRNA Sample Preparation kit (Illumina, San Diego, CA, USA) per the manufacturer’s instructions. Libraries were evaluated with an Agilent 2100 bioanalyzer and quantified by qPCR using the CFX96 Real Time System (Bio-Rad, Hercules, CA, USA), then sequenced on a NextSeq500 sequencer (Agilent, Santa Clara, CA, USA) with a paired-end 75 bp plus single 8 bp index read run. The raw data from the RNA analysis were converted into sequence data and stored as FASTQ files. Differentially expressed genes (DEGs) were determined based on an absolute fold change (FC) greater than 1.4 and a *p* < 0.05. Reactome Pathway analysis was performed to identify significant DEGs using Enrichr (2016) [21]. The 3 animals per group were pooled into 1 biological replicate.

### 2.13. Quantitative PCR (qPCR)

Twenty days post-administration, total RNA was extracted from mice cochleae using TRIzol™ reagent (Thermo Fisher Scientific Korea Ltd., Seoul, Republic of Korea) per the manufacturer’s protocol. Briefly, 1 μg of total RNA was reverse-transcribed using the RevertAid First Strand cDNA Synthesis Kit (Thermo Fisher Scientific Korea Ltd., Seoul, Republic of Korea). Relative mRNA expression was measured by qPCR, normalized to *β-actin*. qPCR reactions contained 5 μL SYBR Select Master Mix (Applied Biosystems, Thermo Fisher Scientific Korea Ltd., Seoul, Republic of Korea), 1 μL cDNA template, 1 μL each of forward and reverse primers (10 pmol each), and 2 μL RNAse-free water. The qPCR parameters were initial denaturation at 95 °C for 5 min, followed by 45 cycles of 95 °C for 15 s, 60 °C for 15 s, and 72 °C for 20 s, with a melting step at 73 °C for 5 min. Gene expression was analyzed using the 2−ΔΔCt method. Primer sequences are listed in Table 1.

### 2.14. Statistical Analyses

Statistical analyses were performed using GraphPad Prism version 5.01 software (GraphPad Software, San Diego, CA, USA). All data are expressed as the mean ± standard error of the mean (SEM). The statistical significance between groups was determined using a paired *t*-test or a one-way repeated measures ANOVA followed by Tukey’s post hoc test. Statistical significance was set at *p* < 0.05.

## 3. Results

### 3.1. CAE’s Efficacy on Otic Hair Cell Protection after NM-Induced Ototoxicity in Zebrafish

**Figure 1:**
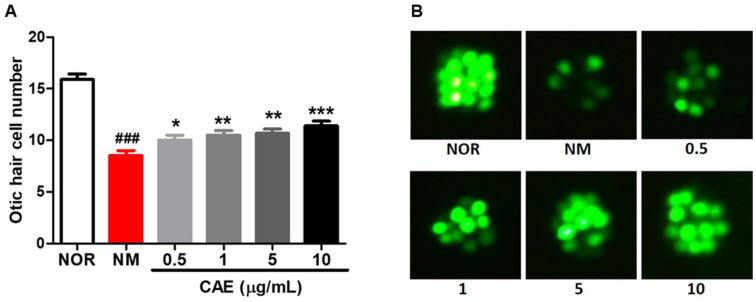
CAE’s efficacy on otic hair cell protection after neomycin-induced ototoxicity in zebrafish model. (**A**) Number of otic hair cells in the untreated group (NM) and the treated groups (0.5, 1, 5, and 10 µg/mL of CAE). (**B**) Fluorescence images of otic hair cells in the normal (NOR), control (NM), and treated groups. Hair cells were stained with YO-PRO-1 at 0.1%. Data are presented as means ± SEM. * *p* < 0.05, ** *p* < 0.01, *** *p* < 0.001 (control vs. treated groups). ^###^ *p* < 0.001 (normal vs. control group). *n* = 10 per group.

The efficacy of CAE on otic neuromast hair cell protection was assessed following exposure to the ototoxic drug neomycin (NM) using zebrafish. Hair cells within the otic (O1) neuromast were damaged after NM exposure (Figure 1). NM exposure significantly reduced (*p* < 0.001) the number of otic hair cells. In contrast, the CAE treatment significantly promoted hair cell protection in a dose-dependent manner (*p* < 0.05, *p* < 0.01, and *p* < 0.001) compared to the control. These findings indicate that CAE is effective in facilitating otic hair cell protection following ototoxicity induced by NM.

### 3.2. EC_50_, LC_50_, and TI Values of CAE in Zebrafish

**Figure 2:**
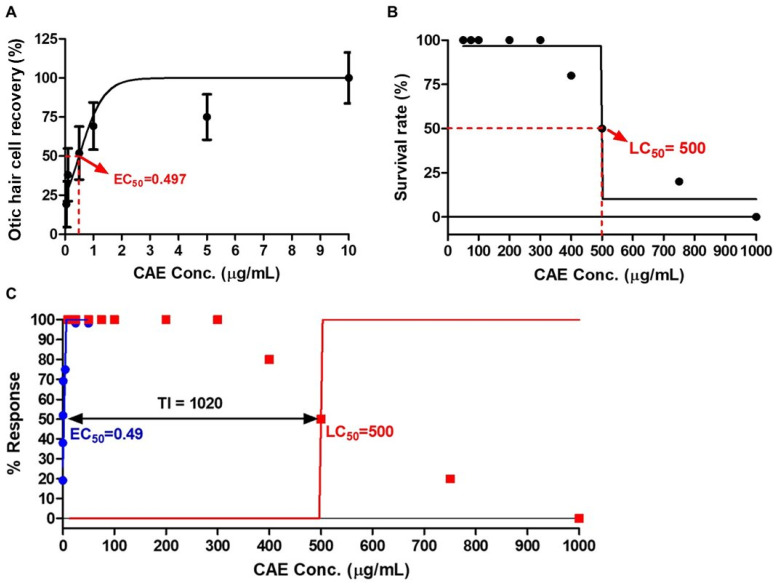
Dose–response curves and therapeutic index of CAE. (**A**) The EC_50_ value of CAE in neomycin (NM)-induced ototoxicity was defined as 0.497 µg/mL. (**B**) The LC_50_ value of zebrafish embryos exposed to CAE for 48 h was defined as 500 µg/mL. (**C**) The therapeutic index (TI) of CAE was calculated to be 1020, indicating a high level of drug safety. Data are presented as means ± SEM. Conc. = concentration; EC_50_ = 50% effective concentration; LC_50_ = 50% lethal concentration. *n* = 10 per group for EC_50_; *n* = 20 per group for LC_50_.

To determine the EC_50_ value of CAE in NM-induced ototoxicity, a dose–response curve was generated for CAE-treated zebrafish at varying concentrations (0.01 to 10 µg/mL). The EC_50_ value of CAE was defined as 0.497 µg/mL (Figure 2A).

To assess the LC_50_ value, mortality rates were examined in zebrafish exposed to CAE at varying concentrations (50 to 1000 µg/mL). The LC_50_ value of CAE was defined as 500 µg/mL (Figure 2B).

The therapeutic index (TI) of CAE was then calculated; TI is defined as the ratio of LC_50_ to EC_50_ (LC_50_/EC_50_). The TI is an index of drug safety, with higher TI values indicating greater safety. The TI of CAE was calculated to be 1020, indicating a high level of therapeutic safety for the use of CAE (Figure 2C).

### 3.3. Toxicity Evaluation of CAE

**Figure 3:**
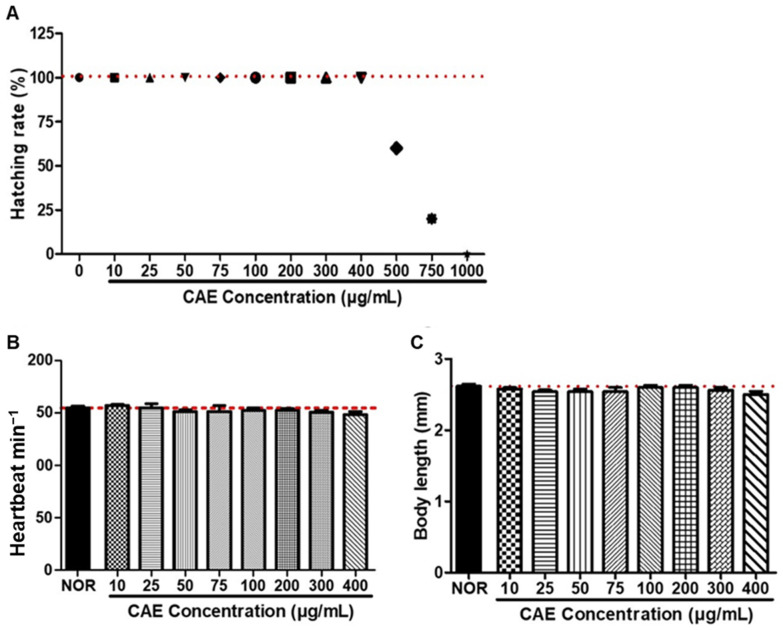
Toxicity evaluation of CAE based on zebrafish embryo testing. Data represent 48 h of exposure. (**A**) Hatching rate of zebrafish embryos exposed to CAE at varying concentrations: 10–1000 µg/mL. (**B**) Heartbeat rate (beats per minute) of zebrafish treated with CAE at varying concentrations: 10–400 µg/mL. (**C**) Body length of zebrafish treated with CAE at varying concentrations: 10–400 µg/mL. Data are presented as means ± SEM. *n* = 20 per group.

To evaluate CAE toxicity in zebrafish embryos, we investigated the hatching rate, heartbeat, body length, and morphological changes in zebrafish embryos after 48 h of exposure (Figure 3). A significant decrease in the hatching rate was observed in zebrafish treated with CAE at concentrations of 500 µg/mL or higher. However, no significant differences were detected between the CAE-treated and non-treated groups at concentrations up to 400 µg/mL (Figure 3A). Additionally, no significant differences were found in the heartbeat and body length of zebrafish treated with CAE compared to the non-treated group at concentrations up to 400 µg/mL (Figure 3B,C). These results indicate that CAE does not exhibit toxicity at concentrations up to 400 µg/mL.

### 3.4. CAE Effects on Auditory Function in NIHL Mouse Model

**Figure 4:**
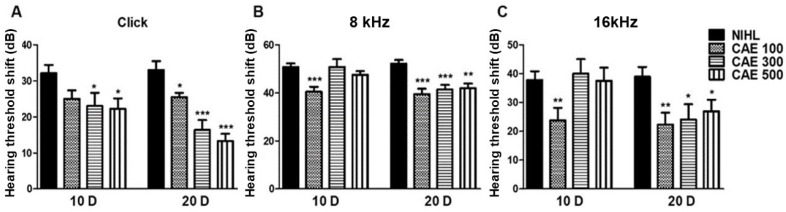
Effects of CAE on auditory function in NIHL mouse model. No-treatment (NIHL) and CAE-treated groups were compared. Auditory brainstem response (ABR) threshold shifts with click stimulus (**A**), 8 kHz tone burst (**B**), and 16 kHz tone burst (**C**) in mouse model at 10 days (10 D) and 20 days (20 D) after noise insult. Data are presented as means ± SEM. * *p* < 0.05, ** *p* < 0.01, *** *p* < 0.001 (NIHL group vs. CAE-treated groups). CAE 100, 100 mg/kg; CAE 300, 300 mg/kg; CAE 500, 500 mg/kg. *n* = 10 per group.

We assessed the impact of CAE on auditory function using the auditory brainstem response (ABR) test (Figure 4). The ABR thresholds prior to noise exposure were within normal ranges across the evaluated frequencies for all mice included in this study. Threshold shifts were elevated on day 1 post-noise exposure. However, 20 days post-administration of CAE treatment at various concentrations, the threshold shifts significantly decreased (*p* < 0.05, *p* < 0.01, and *p* < 0.001) compared to the control group in response to click, 8 kHz, and 16 kHz stimuli. Additionally, CAE exhibited a dose-dependent efficacy for click thresholds (Figure 4A). In contrast, at 8 and 16 kHz, the 100 µg/mL CAE treatment demonstrated a greater effect than higher concentrations (Figure 4B,C).

### 3.5. CAE Alleviation of Cochlear Hair Cell Damage in NIHL Mice

**Figure 5:**
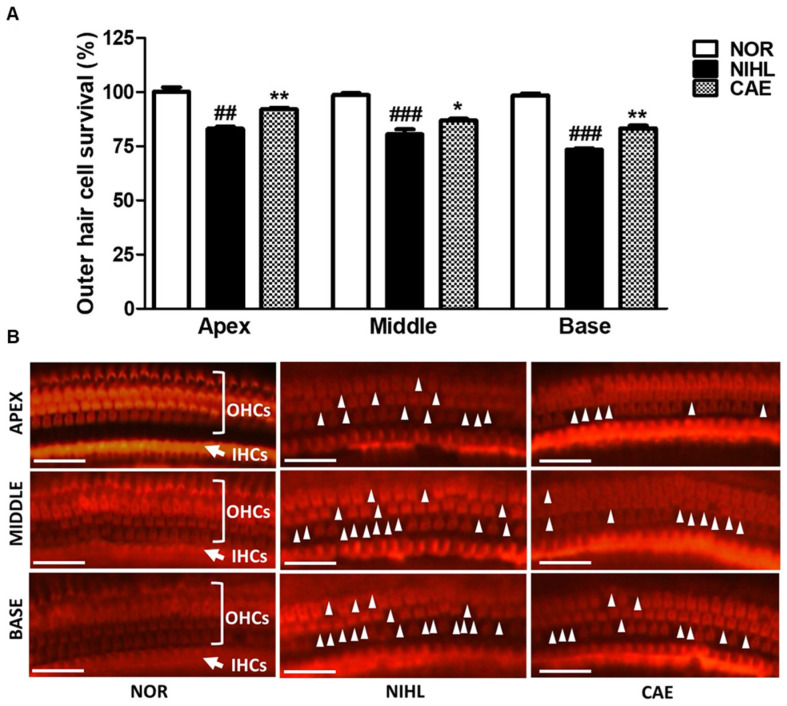
CAE alleviated cochlear hair cell damage in NIHL mice. (**A**) Outer hair cell (OHC) survival in 1 mm segments from the apex, middle, and base of the cochlea (*n* = 6 per group). (**B**) Fluorescence images of the outer (OHC) and inner (IHC) hair cells at the apex, middle, and base of the cochlea by Rhodamine phalloidin staining. Scale bar = 50 µm. ^##^ *p* < 0.01, ^###^ *p* < 0.001 (normal group vs. NIHL group). * *p* < 0.05, ** *p* < 0.01 (NIHL group vs. CAE-treated group). NOR = normal. CAE 100, 100 mg/kg. White triangles indicate the locations where the loss of outer hair cells occurred.

Given that CAE at 100 mg/kg demonstrated superior efficacy in improving hearing function, we evaluated its effects on cochlear hair cells at the apex, middle, and base following noise-induced damage (Figure 5). Noise exposure resulted in a significant loss of outer hair cells (OHCs) in the NIHL group (*p* < 0.01, *p* < 0.001), particularly in the base of the cochlea (Figure 5A). In contrast, CAE administration after NIHL substantially protected hair cells from noise-induced damage (Figure 5B). As shown in Figure 5A, the quantification of OHCs revealed significantly higher numbers of OHCs (*p* < 0.05, *p* < 0.01) in the CAE-treated mice compared to the untreated NIHL group. These results indicate that CAE has therapeutic potential in hair cell protection.

### 3.6. Differential Gene Expression by CAE Treatment in the Cochlea of NIHL Mice

**Figure 6:**
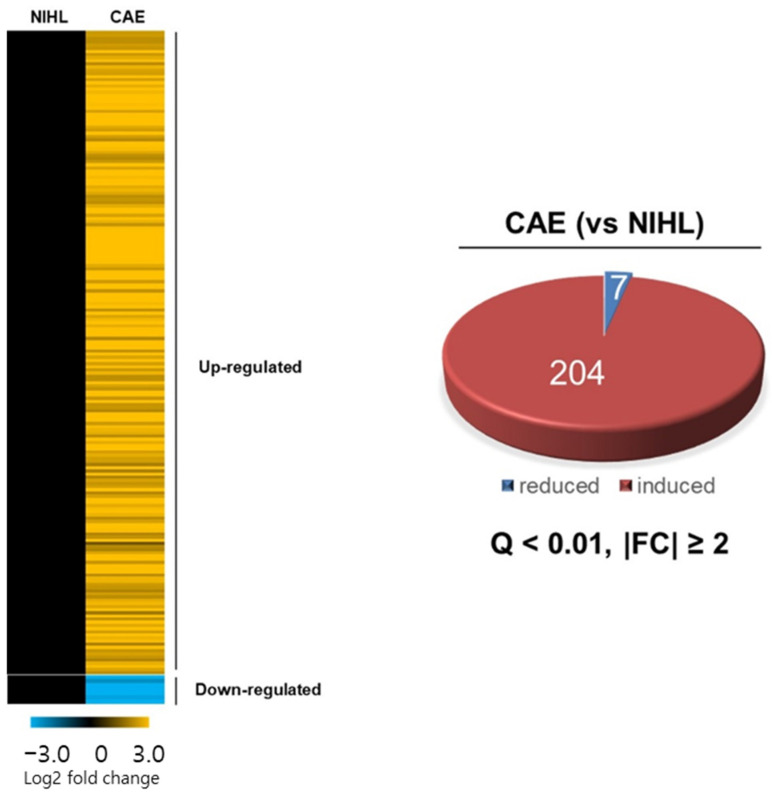
Differential gene expression induced by CAE treatment in the cochlea of NIHL mice (*n* = 3 per group) using Reactome Pathway analysis. Heat map based on RNA-seq analysis of gene expression in the mouse cochlea and Venn diagram showing the overlap of RNA-seq results for the regulated gene set of CAE vs. NIHL group. Genes were categorized into CAE-induced and CAE-repressed groups. Of the total genes, 211 were significantly altered by CAE treatment (false discovery rate (FDR) adjusted Q < 0.01, |fold change| ≥ 2.0).

To further investigate the protective mechanism of CAE in noise-induced hair cell damage, we conducted a transcriptome analysis to identify genes affected by CAE. RNA sequencing was performed to monitor differential gene expression in the cochlea of NIHL mice treated with CAE 100 mg/kg. Of the total genes expressed in the cochlea, 211 genes were differentially expressed (with false discovery rate (FDR) adjusted Q < 0.01, |fold change| ≥ 2.0) by the CAE treatment. Among these, 204 genes were up-regulated and 7 genes were down-regulated (Figure 6).

**Figure 7:**
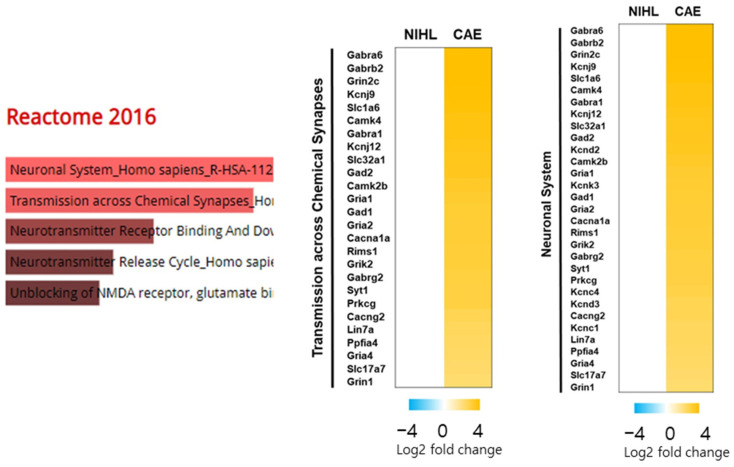
Functional categorization of genes up-regulated by CAE 100 mg/kg using Reactome Pathway analysis (*n* = 3 per group). Heat map generated from RNA-seq data showing gene sets involved in transmission across chemical synapses (*p* value 8.74 × 10^−21^; Q value 1.21 × 10^−18^; Enrichment score 101.5087) and the neuronal system (*p* value 2.92 × 10^−22^; Q value 8.09 × 10^−20^; Enrichment score 110.3959).

A Reactome Pathway analysis was performed to identify the functional pathways (Figure 7). We focused on the genes up-regulated by the CAE treatment that were enriched in the transmission across chemical synapses (*p* value 8.74 × 10^−21^; Q value 1.21 × 10^−18^; Enrichment score 101.5087) and the neuronal system (*p* value 2.92 × 10^−22^; Q value 8.09 × 10^−20^; Enrichment score 110.3959) pathways.

### 3.7. CAE Effects on Neurotransmitter Synthesis, Secretion, Transport, and Neuronal Survival Gene Expression in NIHL Mice

**Figure 8:**
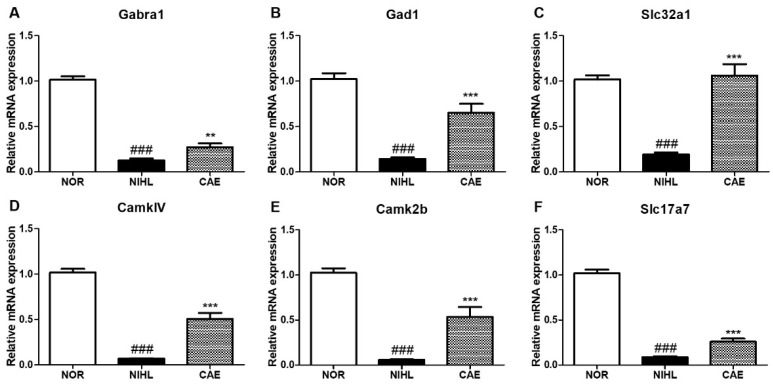
Changes in the expression of neuronal function-related genes in NIHL mice treated with CAE 100 mg/kg. Gene expression changes were evaluated by qPCR 20 days after noise insult. The effects of CAE treatment on genes related to inhibitory synaptic transmission (**A**–**C**), neuronal survival (**D**,**E**), and synaptic function (**E**,**F**) are shown. Data are presented as means ± SEM. ^###^ *p* < 0.001 (NOR group vs. NIHL group); ** *p* < 0.01, *** *p* < 0.001 (NIHL group vs. CAE-treated group). NOR = normal.

To validate the RNA-seq results, we examined the effect of NIHL on the expression of genes affected by the CAE treatment (Figure 8). NIHL mice showed decreased expression of genes related to neuronal function compared to normal mice. Specifically, the expression of genes related to inhibitory synaptic transmission (*Gabra1*, *Gad1*, and *Slc32a1*), neuronal survival (*CaMK2b* and *CaMKIV*), and synaptic function (*CaMK2b* and *Slc17a7*) were significantly decreased by NIHL, suggesting that noise exposure reduces markers of neuronal function and survival. Moreover, these genes’ expression was significantly increased by CAE compared to the NIHL group. These results corroborate the RNA-seq findings and suggest that the protective mechanism of CAE may involve the enhancement of neuronal function and survival.

## 4. Discussion

In the present study, we evaluated the potential efficacy of CAE, given that certain species of the genus Castanopsis are known to be rich in polyphenols such as flavonoids and have demonstrated activity against oxidative stress, inflammation, and apoptosis [16,17,18,19], which are key pharmacological targets in SNHL. However, there are no studies of Castanopsis species effects on SNHL. Specifically, CAE has not been reported to have any pharmacological activity. Therefore, we assessed the efficacy of CAE in two models of SNHL: one induced by ototoxic agents and the other by noise exposure. Additionally, we sought to elucidate its possible mode of action.

Protecting sensory hair cells has become a crucial target in the treatment of SNHL, as the condition often results from hair cell loss [6,22,23]. The zebrafish lateral line has gained prominence as a model system due to its morphological and physiological similarities to mammalian cochlear hair cells. This model is valuable for investigating the underlying mechanisms of pathology and for easily screening new candidates with potential activity as SNHL treatments [24,25]. In this study, we evaluated the effect of CAE on otic hair cell protection following ototoxicity induced by neomycin. Aminoglycoside ototoxicity, such as that caused by neomycin, is well known to damage sensory hair cells through mechanisms like those observed in NIHL [26]. Our results demonstrated that CAE significantly promotes otic hair cell protection, with an EC_50_ value of 0.497 µg/mL. Additionally, our investigation into the toxicity of CAE revealed that it is a safe drug, as indicated by its high therapeutic index (TI) value of 1020.

Because noise exposure is one of the most prevalent forms of SNHL [23], we evaluated CAE’s activity on auditory function in an NIHL mouse model. It is well known that NIHL causes an increase in auditory thresholds and alterations in the waveforms of the ABR, indicating a reduced response in different regions of the auditory pathway [27]. Our results demonstrated that different doses of CAE were effective at the click, 8 kHz, and 16 kHz frequencies, as evidenced by decreased hearing thresholds in the ABR. CAE significantly improved auditory function after 20 days of treatment and also exhibited a protective effect on sensory hair cells in the apex, middle, and base of the cochlea, as shown by rhodamine phalloidin staining. Specifically, the effect of CAE was greater with the click stimulus. The click stimulus contains several frequencies along the cochlear traveling wave [28]. In contrast, tone bursts specifically represent 8 and 16 kHz. Our findings in the click stimulus may also be related to higher frequencies not considered in this study. As shown in the ABR data, the effect of CAE was greater at 16 kHz tone burst thresholds than at 8 kHz, which could support the hypothesis of its efficacy at higher frequencies. This study provides compelling evidence for the potential of CAE as a treatment for SNHL.

To elucidate the mode of action of CAE, we assessed the differential expression of genes in NIHL mice following CAE treatment using RNA-seq. Among the genes affected by CAE, we focused specifically on those involved in hearing impairments. The up-regulated genes of interest were enriched in pathways related to chemical synaptic transmission and the neuronal system.

Interestingly, we observed that the enriched genes were involved in neuronal function, neuronal survival, and GABA inhibitory mechanisms. CAE up-regulated genes involved in GABAergic inhibition, including GABA receptor genes (*Gabrd*, *Gabra6*, *Gabrb2*, *Gabra1*, *Gabrg2*); glutamate decarboxylase genes (*Gad1*, *Gad2*), which catalyze the production of GABA from L-glutamic acid; and a GABA transporter-related gene (*Slc32a1*). Impairments in GABA-mediated synaptic transmission have been implicated in the pathogenesis of auditory disorders [29]. A significant reduction in GABA receptors has been reported in both noise-induced and age-related hearing loss, and increasing the expression of GABA receptors has been suggested as a potential target for NIHL treatment to enhance auditory function [30,31,32]. Additionally, *Gad1* expression has been reported to decrease in the cochlear nucleus and the inferior colliculus in hearing loss models [33].

Furthermore, CAE up-regulated genes related to synaptic function, such as *Slc17a7* and *CaMK2b*. Synaptic transmission at cochlear hair cells and spiral ganglion neurons is crucial for normal hearing function. The *Slc17a7* gene, also known as *Vglut1*, encodes the vesicular glutamate transporter 1 (VGLUT1) protein, which is required for the transmission of sound-induced signals from the cochlea to the central auditory pathway. Cochlear damage induced by noise or ototoxic drugs results in decreased *Vglut1* expression [34,35]. The *CaMK2b* gene encodes the calcium/calmodulin-dependent protein kinase II beta (CaMKIIβ) protein. Genes related to calcium homeostasis, such as *CaMK2b*, are important in ototoxic insults, including cisplatin exposure, which leads to the decreased expression of genes related to neurotransmitter secretion and transport [36]. The up-regulation of these genes has been associated with increased survival of cochlear hair cells and spiral ganglion neurons after cisplatin ototoxicity, making this an interesting pharmacological approach [36].

As mentioned previously, SNHL results from damage to otic hair cells and cochlear spiral ganglion neurons. In vivo studies have demonstrated that it is possible to counteract this damage by enhancing survival mechanisms, such as growth factors [34]. Both Ca^2+^/calmodulin-dependent protein kinases II and IV (*CaMKII* and *CaMKIV*) have shown survival-promoting effects on spiral ganglion neurons in vitro [37,38,39]. CAE up-regulated both *CaMKII* and *CaMKIV*, suggesting that its mechanism of action might also involve improved survival of cochlear spiral ganglion neurons.

To validate the RNA-seq results, we selected six neuronal function-related genes identified in the cochlea of CAE-treated mice for qPCR analysis. The target genes were related to GABA inhibitory action (*Gabra1*, *Gad1*, and *Slc32a1*), neuronal survival (*CaMK2b* and *CaMKIV*), and synaptic function (*CaMK2b* and *Slc17a7*). As expected, NIHL mice showed significantly decreased mRNA expression of genes related to neuronal function and survival compared to normal mice, whereas the CAE treatment significantly increased the mRNA levels of these genes.

## 5. Conclusions

Our data suggest that auditory function improvement and protection of sensory cells by CAE might be explained through increased genes related to synthesis, secretion, and transport of neurotransmitters and neuronal survival. In addition, this study provides more evidence about changes in genes related to neuronal function in the mice cochlea after noise-induced hearing loss. Thus, we propose a therapeutic approach based on recent perspectives to treat hearing impairments by improving neuronal function and survival. However, a limitation of this study is the lack of sufficient evidence to support proceeding to clinical trials, necessitating additional research on toxicity, pharmacokinetics, pharmacodynamics, and dosage optimization. Another limitation in this study is the lack of data demonstrating the difference in gene expression between the normal and NIHL groups, which represents an opportunity for further studies. It would be necessary to conduct a timeline study of the up-regulated and down-regulated genes in NIHL compared to normal mice to better understand the optimal intervention with natural products, mainly to translate these studies into clinical trials. Additionally, in subsequent studies, we will characterize this extract to find the main compounds responsible for the therapeutic efficacy of CAE and their potential synergistic effects.

## Figures and Tables

**Table 1:** Primers for qPCR.

Gene Name	Forward Sequence	Reverse Sequence
*β-Actin*	GAAGAGCTATGAGCTGCCTGA	TGATCCACATCTGCTGGAAGG
*Gabra1*	AATGGGCGGATTGGTGTC	TCATCTTGGGAGGGCTGT
*Gad1*	GCCTGGAAGAGAAGAGTCGT	TCCCCGTTCTTAGCTGGAAG
*Slc17a7*	TGGCTGTGTCATCTTCGTGAGG	TTGCCAGCCGACTCCGTTCTAA
*Camk4*	GGAGAAGGGATACTACAGTGAGC	CTGGTTTGAGGTCACGATGGAC
*Camk2b*	CCTACGGCAAACCTGTGGACAT	GCCTTGATCTGCTGGTACAGCT
*Slc32a1*	GGCTGGAACGTGACAAATGCCA	TACAGGCACGCGATGAGGATCT

## Data Availability

Data are contained within this article.
